# Suite of Biochemical and Cell-Based Assays for the Characterization of Kirsten Rat Sarcoma (KRAS) Inhibitors and Degraders

**DOI:** 10.1021/acsptsci.4c00450

**Published:** 2024-12-02

**Authors:** Medhanie Kidane, Rene M. Hoffman, Jennifer K. Wolfe-Demarco, Ting-Yu Huang, Chi-Ling Teng, Saheli Samanta, Luis M. Gonzalez Lira, Jennifer Lin-Jones, Gabriel Pallares, Jane E. Lamerdin, Nicole B. Servant, Chun-Yao Lee, Chao-Tsung Yang, Jean A. Bernatchez

**Affiliations:** † Research and Development and Technology Transfer, Eurofins DiscoverX, LLC, 11180 Roselle Street Suite D, San Diego, California 92121, United States; ‡ Research and Development, Eurofins DiscoverX Products, LLC, 42501 Albrae Street, Fremont, California 94538, United States; § Eurofins Panlabs Discovery Services Taiwan, Ltd., 25 Wugong Sixth Road, Wugu District, New Taipei City 24891, Taiwan

**Keywords:** KRAS, dissociation constant, target engagement, cell signaling, targeted protein degradation

## Abstract

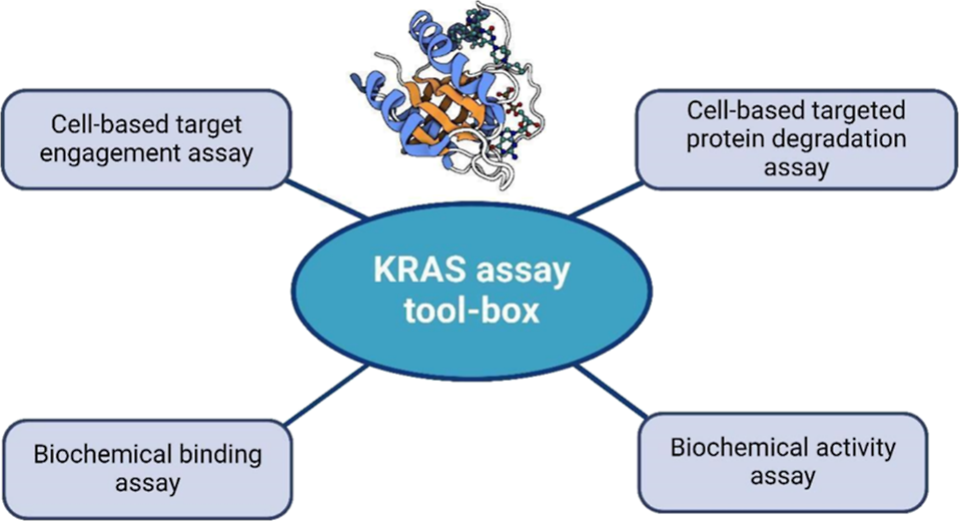

KRAS is an important oncogenic driver which is mutated in numerous cancers. Recent advances in the selective targeting of KRAS mutants via small molecule inhibitors and targeted protein degraders have generated an increase in research activity in this area in recent years. As such, there is a need for new assay platforms to profile next generation inhibitors which improve on the potency and selectivity of existing drug candidates, while evading the emergence of resistance. Here, we describe the development of a new panel of biochemical and cell-based assays to evaluate the binding and function of known chemical entities targeting mutant KRAS. Our assay panels generated selectivity profiles and quantitative binding interaction dissociation constants for small molecules and degraders against wild type, G12C, G12D, and G12V KRAS, which were congruent with published data. These assays can be leveraged for additional mutants of interest beyond those described in this study, using both overexpressed cell-free systems and cell-based systems with endogenous protein levels.

Kirsten rat sarcoma (KRAS) is a small GTPase that plays an important role in the regulation of biochemical signaling for cellular growth, proliferation, differentiation, and migration. Through its cycling between a guanosine triphosphate (GTP)-bound “on” state and a guanosine diphosphate (GDP)-bound “off” state, the protein can respond to extracellular signaling and propagate a biochemical signal via interaction with downstream effectors.^1^ Activating mutations of KRAS are among the most common driver mutations in human cancers, and frequently alter the protein’s structure such that GTPase activating proteins (GAPs) are less able to hydrolyze GTP to GDP when bound to KRAS. This in turn biases mutant KRAS to adopt an “on” confirmation, even in the absence of an extracellular signal, thereby driving aberrant downstream cellular signaling and tumor formation.^1^ A majority of KRAS mutations occur at three codon positions (G12, G13, Q61), though mutations at other positions in KRAS also can lead to the deregulation of its activity.^2^ These mutations have a high occurrence in lung,^3^ colorectal^4^ and pancreatic cancers.^5^

The prevalence of KRAS mutations has made the protein a prime target for oncology drug discovery for decades. However, KRAS was thought to be undruggable until the recent development of KRAS(G12C) inhibitors,^6−9^ including MRTX849 (adagrasib)^10,11^ and AMG510 (sotorasib)^12,13^ that have reached the clinic. Efforts are being made to expand inhibitor space to other mutants beyond KRAS(G12C).^14−24^ Furthermore, manipulation of the ubiquitin–proteasome system to achieve targeted degradation of hyper-active or overabundant proteins that cause diseases has become a new approach for therapeutic discovery, including for KRAS mutants.^25^

The recent clinical success of the KRAS(G12C) inhibitors MRTX849^10^ and AMG510^12^ has ignited extensive interest to adapt this modality into bifunctional degraders that could reduce hyperactive KRAS mutant proteins for cancer therapeutics. Commercially available LC2 is a mutant selective KRAS degrader that consists of MRTX849 chemically linked to a ligand for the von Hippel Lindau (VHL) E3 ligase.^10,25,26^ Using a Western blot approach, it has been reported that LC2 induces selective degradation of KRAS(G12C) protein with DC_50_ around 0.25–0.76 μM and *D*_max_ around 75%–90% among three different cell lines without causing degradation of other forms of KRAS proteins in cancer cell lines. The degradation of the KRAS(G12C) protein was shown to lead to a decrease of MAPK signaling in these cells and consequently growth suppression.^25,27^

Accurate measurement of the dissociation constants (*K*_D_s) for KRAS inhibitors such as the KRAS G12D-selective inhibitor MRTX1133^15,28,29^ has sometimes been challenging. This is particularly the case when the affinity of the ligand for the target is very high and the *K*_D_ for the interaction is below the detection limit of the technology used for the measurement, as was the case when surface plasmon resonance (SPR) was used to interrogate the binding affinity of MRTX1133 for KRAS(G12D).^15^*K*_D_ values for high-affinity ligands have been successfully determined in the past for kinases^30−33^ using an assay system that tags the kinase with a DNA probe that can detect picomolar quantities of protein via qPCR. This allows for the accurate determination of the true thermodynamic dissociation constant for a ligand without hitting the tight-binding limit of the assay for picomolar affinity ligands.^33^

Traditional assays for protein degradation and turnover, such as cell viability assays, ELISAs and Western blots, are laborious and time-consuming.^34−37^ As such, these traditional assays are also difficult to implement in compound screening cascades with automation for hit-to-lead campaigns. Hence, a homogeneous cell-based assay without the need for reliable antibodies is desirable for the discovery of new protein degraders in a high throughput manner. Despite the common use in drug discovery of cell lines that overexpress a target protein, these systems sometimes have limitations when accurately determining the potencies of many degraders.

The cellular thermal shift assay (CETSA) was developed to assess drug–target interaction by measuring changes in a protein’s thermal stability upon compound binding in a cellular environment. Despite the assay’s advantage of not requiring compound modifications, the traditional antibody-based detection methods of CETSA have hindered its application to high throughput library screening. The recent development of reporter-based systems that build on the heterologous expression of the targets with appended small reporter tags has greatly improved the throughput of the assay. However, the potential artifacts of transgene overexpression may confound the interpretation of the target engagement results and demand assays that allow the detection of interactions between drugs and targets expressed endogenously in a more native cellular environment.^38^

To better characterize KRAS-targeting compounds for drug discovery efforts in this space, we developed both biochemical and cell-based assays to assess target engagement by small molecules, and functional assays to assess the effect of KRAS degraders on endogenous protein levels in relevant cancer cell models.

The biochemical competition-based binding assay described in this paper for KRAS is based on the qPCR-amplicon tagging technology mentioned above. In this assay, a DNA-tagged protein of interest is collected as part of a mammalian cell protein extract, and then incubated in the presence of a capture ligand immobilized on magnetic beads. The KRAS-binding warhead of the immobilized capture ligand is taken from a compound series that has been shown to engage the switch II binding pocket of KRAS, which has been a successful strategy for the allosteric inhibition of this target’s activity.^15^ The aforementioned components are further incubated in the presence of a dimethyl sulfoxide (DMSO) vehicle control or a test competitor compound. If the test compound can compete with the immobilized capture ligand for binding to the target protein, less protein is enriched on the bead in this case than in the presence of a vehicle control. After quantification of the protein that was captured on the bead via qPCR, a lower qPCR signal would be observed for a compound that was able to compete with the immobilized capture ligand for binding to the target protein.

In contrast with other binding technology systems, such as nuclear magnetic resonance (NMR) and electrophoretic mobility shift assays (EMSAs), the proposed biochemical binding assay system is 384-well plate compatible, can be used for large library profiling and is automated with liquid handlers and washer systems. Also, unlike these aforementioned other binding assay systems and the fluorescence polarization (FP), microscale thermophoresis (MST) and surface plasmon resonance (SPR) methods, our proposed assay system does not require previous protein purification to perform the assay. Furthermore, the low amount of protein used in our biochemical binding assay allows for the accurate measurement of high affinity ligands of the target protein (with *K*_D_ values as low as 100 pM and potentially even lower) and represents a technical improvement over other biochemical binding assay systems. For these reasons, the biochemical binding assay system described in this paper represents a new, useful tool for high-throughput screening of small molecule binders of KRAS(wild-type, WT) and mutant KRAS.

The nucleotide exchange assay (NEA) is a widely used technique to study RAS functional activity between the “off” (GDP) and “on” (GTP) states of the protein; this process is mediated by guanine nucleotide exchange factors (e.g., SOS1).^39,40^ To complement the inhibitor data collected from the developed biochemical binding assays, we performed activity assays where fluorophore-labeled GDP was used and GDP to GTP exchange across KRAS(WT), KRAS(G12C), KRAS(G12D), and KRAS(G12V) was directly measured through time-resolved fluorescence energy transfer (TR-FRET),^41^ as previously described.^40^ This assay is a standard literature method for determining the effect of KRAS inhibitors on nucleotide exchange, and acts as a control assay for our other developed biochemical and cell-based assays in this study to ensure concordance of our developed assay systems with an established protocol. Furthermore, this nucleotide exchange assay system captures the functional effect of the compounds tested in this study in a biochemical system, which is complementary to the biochemical binding assays we developed for this study.

To meet the need for homogeneous cell-based degradation assays, a new type of cell line was developed based on the enzyme fragment complementation (EFC) method to capture endogenous expression of target proteins. EFC is a homogeneous, robust and scalable detection assay system that enables the measurement and ranking of ligand potencies, discovery of the mechanism of action (MOA) of a compound, the performance of functional screens, and identification of novel compounds that engage the target.^42−45^ This approach is based on two recombinant β-galactosidase (β-gal) enzyme fragments that act as an enzyme acceptor (EA) and an enzyme donor (ED). Separately, the fragments are inactive, but when combined, they form an active β-gal enzyme that hydrolyzes its substrate to produce a chemiluminescence signal.^42^ The ED fragment is relatively small (42 amino acids) and can be easily knocked into an endogenous target locus in the cell model of choice to create an ED-target fusion protein that is expressed at physiologically relevant levels. In contrast to another widely used reporter tag with lysines,^46^ the ED fragment contains no lysines, which are residues that can become covalently linked to ubiquitin.^42,47^ Thus, no proteins are artificially degraded due to the introduction of the ED fragment. The endogenous expression levels of ED-target fusion proteins more accurately reflect physiologically relevant protein levels than overexpression systems and thus allow for a more biologically relevant potency assessment of protein degraders. Furthermore, such cell lines offer a great opportunity to investigate target engagement in a native cellular environment where target proteins are expressed at endogenous levels, a model different from commonly used heterologous expression systems. In addition, the InCELL workflow adapts a protocol in which compounds are incubated with live cells as opposed to traditional assays that are carried out with purified target proteins or cell lysates. This workflow further addresses and monitors the cellular permeabilities of the compounds.^48,49^

In this study, we created a biochemical and cell-based assay screening pipeline for the characterization of KRAS inhibitors and degraders using newly developed biochemical binding, cell-based target engagement and cell-based targeted protein degradation assays and an established biochemical nucleotide exchange assay from the scientific literature. Each of these assay systems were included in the study to characterize compounds at the level of binding to KRAS(WT) and KRAS mutants in both a cell-free and cellular background, to assess the functional effect of compounds on KRAS(WT) and mutant KRAS nucleotide exchange in a biochemical system, and to assess the effect of a KRAS degrader on KRAS protein levels in a cellular background. The known biochemical nucleotide exchange assay was included in the study as a point of reference for the newly developed assays, and to assess the ability of the new assays to match existing selectivity and potency trends seen in the nucleotide exchange assay. These assays are complementary to each other and provide a global view of the in vitro parameters of inhibition or protein degradation for the compounds tested in this study.

The biochemical competition binding assays were performed using KRAS(WT), KRAS(G12C), KRAS(G12D) and KRAS(G12V) NFκB DNA-binding domain fusions tagged with qPCR amplicons to detect the protein in the assay readout. We were able to quantitatively measure *K*_D_ values for MRTX1133 and other KRAS-targeting compounds across the constructs tested and were able to recapitulate published rank order selectivity for these small molecules.

In addition, three KRAS cell lines (KRAS(G12C), KRAS(G12D) and KRAS(WT)) were generated by knocking in an ED fragment (and the relevant mutation in KRAS) into the *KRAS* locus by CRISPR technology. We demonstrated that these cell lines are excellent assay tools for investigating protein degraders and inhibitors. First, we showed their utility in functional testing of protein degradation by demonstrating that the LC2 PROTAC (proteolysis-targeting chimera) selectively induces protein degradation in KRAS(G12C) cells and not in KRAS(WT) and KRAS(G12D) cells, matching the published selectivity of the compound for KRAS(G12C).^25^ LC2 has been shown to selectively bind KRAS(G12C) through its MRTX849 warhead^10^ and to recruit the E3 ligase VHL via a VH032 ligand^26^ to ubiquitinate and ultimately degrade KRAS(G12C).^25^ Second, we adapted an InCELL Pulse Target Engagement workflow^48,49^ for these cell lines and demonstrated that they are useful to interrogate the direct binding between KRAS mutants and their specific inhibitors and gained rank order information for the affinity of these inhibitors to their targets.

Together, these assays provide a framework for the interrogation of the binding and functional parameters of small molecule inhibitors and targeted protein degraders of KRAS and its mutants and can be applied to the characterization of new chemical matter being developed against mutant forms of the protein.

## Results

### Biochemical Binding Assays to Determine Quantitative Affinities of KRAS Switch II Pocket Binders

**Figure 1:**
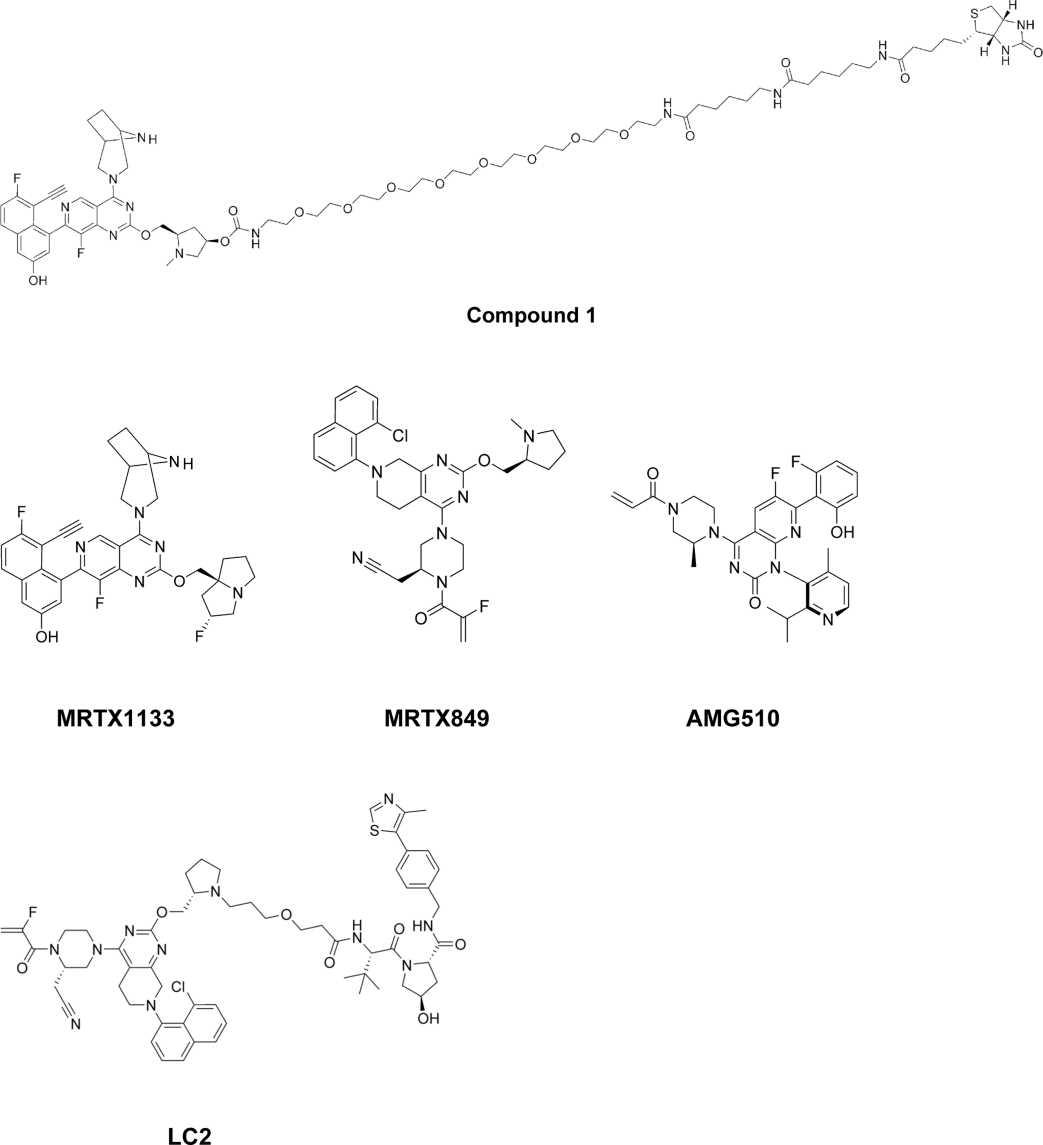
Chemical structures of compounds used in this study. (A) Biotin-conjugated capture ligand for the competitive binding assay. (B) KRAS switch II pocket-binding small molecule inhibitors. (C) KRAS heterobifunctional degrader.

**Figure 2:**
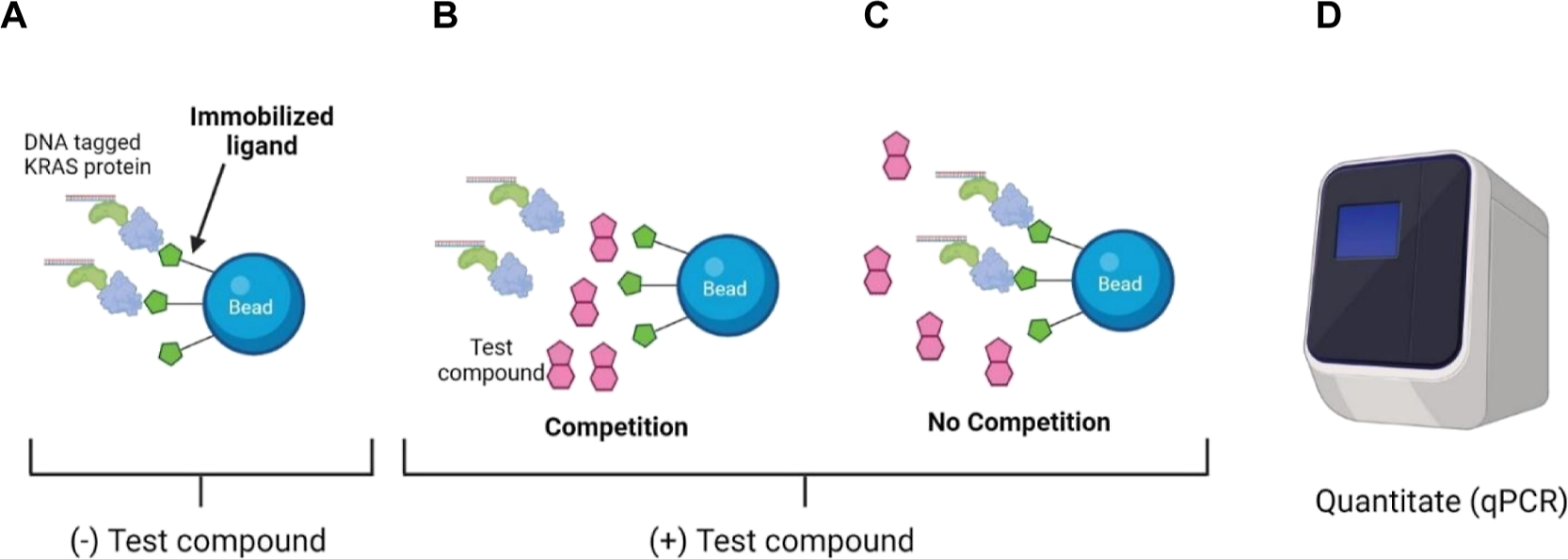
Overview of the biochemical competition binding assay principle. (A) Engagement of the DNA tagged KRAS protein with the immobilized ligand on the bead (bait molecule) in the absence of a test compound and the presence of the DMSO vehicle control is shown. Following washing of the beads and elution of KRAS protein captured on the beads, a high protein concentration is expected in the eluate. (B) In the presence of a test compound that competes with the bait molecule for binding to KRAS, a reduced amount of target protein is captured on the bead. Following washing of the beads and elution of residual KRAS protein, the expected protein concentration in the eluate is lower. (C) In the presence of a test compound that does not compete with the bait molecule for binding to KRAS, a high amount of target protein is captured on the bead. Following washing of the beads and elution of residual KRAS protein, the expected protein concentration in the eluate is high. (D) At the end of the assay, a qPCR reaction is conducted to quantify the amount of amplicon tagged KRAS protein present in the eluate.

**Figure 3:**
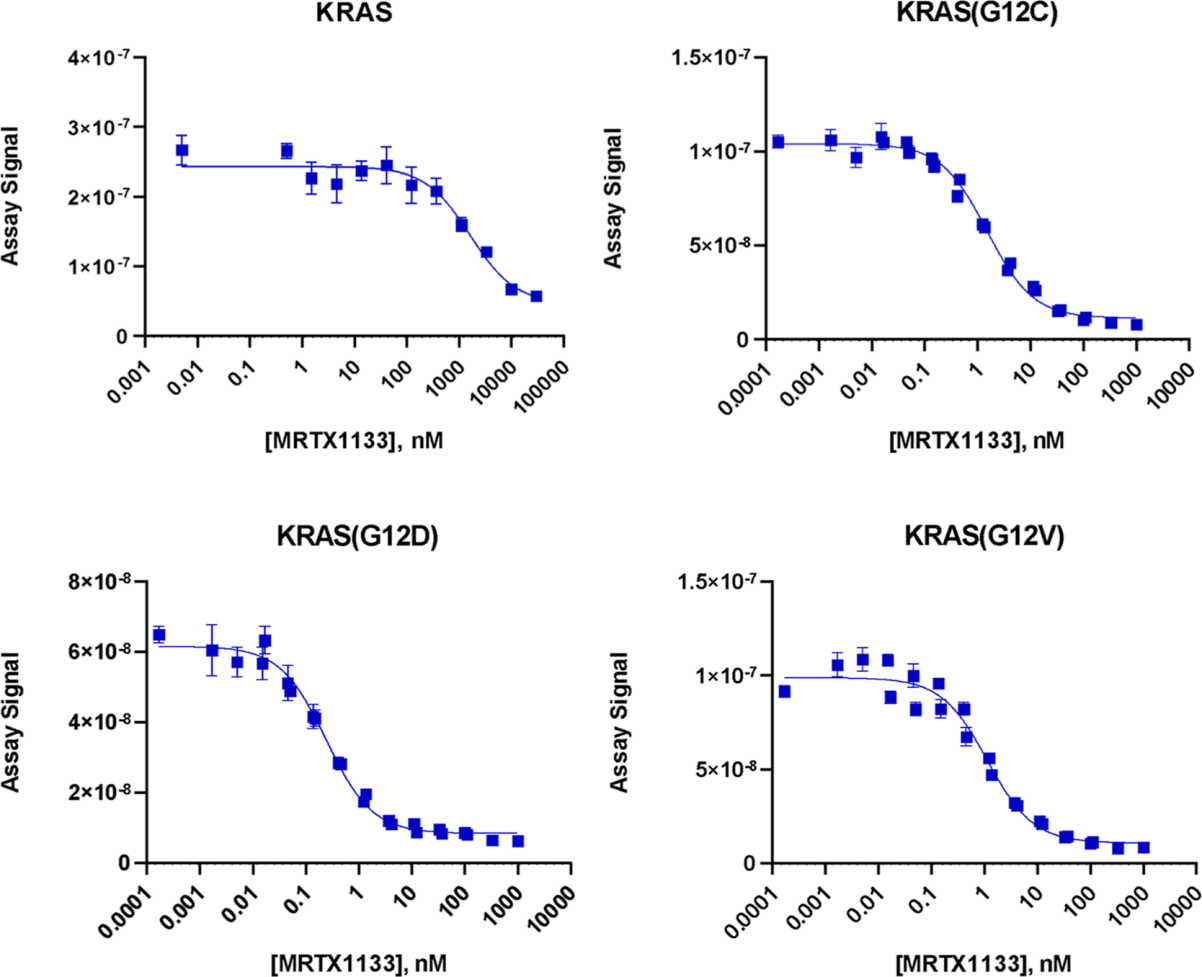
Sample binding curves for MRTX1133 engagement with WT and mutant KRAS in the developed biochemical binding assay. Shown are representative curves for the fitting of qPCR quantification data for recovered KRAS protein in the assay eluate for escalating concentrations of MRTX1133. Error bars on data points represent the standard deviation, *n* = 4 for KRAS(WT), *n* = 8 for KRAS(G12C), KRAS(G12D) and KRAS(G12V).

To provide a new platform to study binding of small molecules to the switch II binding pocket of WT and mutant KRAS proteins, we developed a set of biochemical competitive binding assays, based on a technology platform that has been previously applied to study ligand binding to kinases.^30−33^ KRAS(WT), KRAS(G12C), KRAS(G12D) and KRAS(G12V) constructs with N-terminal fusions of the DNA binding domain of NFκB^50^ were transiently transfected in HEK293 cells. Protein extracts containing these constructs were harvested, then mixed with a DNA probe that anneals with the NFκB fusion domain of the constructs and which contains a qPCR amplicon for protein detection. Finally, the protein extracts were incubated with magnetic beads baited with the biotin-small molecule conjugate compound 1, which contains a ligand moiety previously described as a switch II pocket binder (Figure 1A).^15^ Incubation of the extract with the baited beads was conducted in the presence of either escalating concentrations of a switch II pocket binder (the competitor compounds MRTX1133,^15,28,29^ MRTX849^10,11^ and AMG510^12,13^) (Figure 1B) or a DMSO vehicle control. Following washing and elution of the residual protein bound to the baited beads, a qPCR reaction was conducted to quantify the protein in the eluate. A scheme showing the binding assay principle is depicted in Figure 2. Curve fitting of the competitor dose response data yielded thermodynamic dissociation constants for the competitor compounds (sample curves for MRTX1133 affinity are shown in Figure 3), as described in the Methods. Importantly, the bait loading on the beads was adjusted so that the *K*_D_(app) ≈ *K*_D_ for the competitor molecules tested, as has been previously described for this type of assay.^33^ The obtained *K*_D_ values for MRTX1133, MRTX849 and AMG510 are shown in Table 1. The calculated *Z*′ values for each of the assays (using MRTX1133 as the control competitor) were 0.50 (KRAS(WT)), 0.58 (KRAS(G12C)), 0.60 (KRAS(G12D)) and 0.58 (KRAS(G12V)), indicating the suitability of these assays for compound screening.

**Table 1:** Thermodynamic Dissociation Constants Measured for KRAS Inhibitors Targeting the Switch II Binding Pocket^a^

protein	binding incubation time (h)	compound name	average *K*_D_ (nM)	number of replicates
KRAS	24	MRTX1133	2560 ± 56	6
		MRTX849	>20,000	6
		AMG510	>20,000	6
KRAS(G12C)	1	MRTX1133	2.35 ± 0.10	12
		MRTX849	9.59 ± 2.09	6
		AMG510	220 ± 47	6
KRAS(G12D)	1	MRTX1133	0.40 ± 0.11	12
		MRTX849	>20,000	6
		AMG510	>20,000	6
KRAS(G12V)	1	MRTX1133	1.72 ± 0.11	12
		MRTX849	>20,000	6
		AMG510	>20,000	6

a Average *K*_D_ values for MRTX1133, MRTX849 and AMG510 binding to WT and mutant KRAS are shown for replicates collected over three independent experiments ± standard deviation.

We measured a *K*_D_ of 400 pM for MRTX1133, a selective KRAS(G12D) noncovalent inhibitor, against the G12D mutant; this is in contrast with the previously estimated *K*_D_ of the compound of ∼0.2 pM, where the predicted *K*_D_ of the compound was below the accuracy limit of the SPR instrument used to measure the dissociation constant.^15^ The measured *K*_D_ values for this compound for the G12C and G12V mutants was higher than the value measured for the G12D mutant (2.35 and 1.72 nM, respectively). As expected, the measured affinity of MRTX1133 in this study was lower for KRAS(WT) (*K*_D_ of 2560 nM). Together, these results highlight the selectivity of MRTX1133 for the G12D mutant over the other two G12 mutants tested and the WT protein. In addition, we provide quantitative *K*_D_ values for MRTX1133 for each of these proteins, with a measured subnanomolar *K*_D_ value for KRAS G12D.

The covalent switch II pocket-binding inhibitor MRTX849^10^ displayed a high level of binding selectivity based on our data: we obtained a *K*_D_ of 9.59 nM for KRAS(G12C), and no binding was observed for MRTX849 against the KRAS WT, G12D and G12V protein at compound concentrations up to 20 μM. As with MRTX849, we also observed very selective binding of AMG510^12^ for the G12C mutant, with a *K*_D_ of 220 nM for this protein, and no binding of AMG510 against WT, G12D and G12V proteins at concentrations up to 20 μM.

### Biochemical Activity Assay

**Figure 4:**
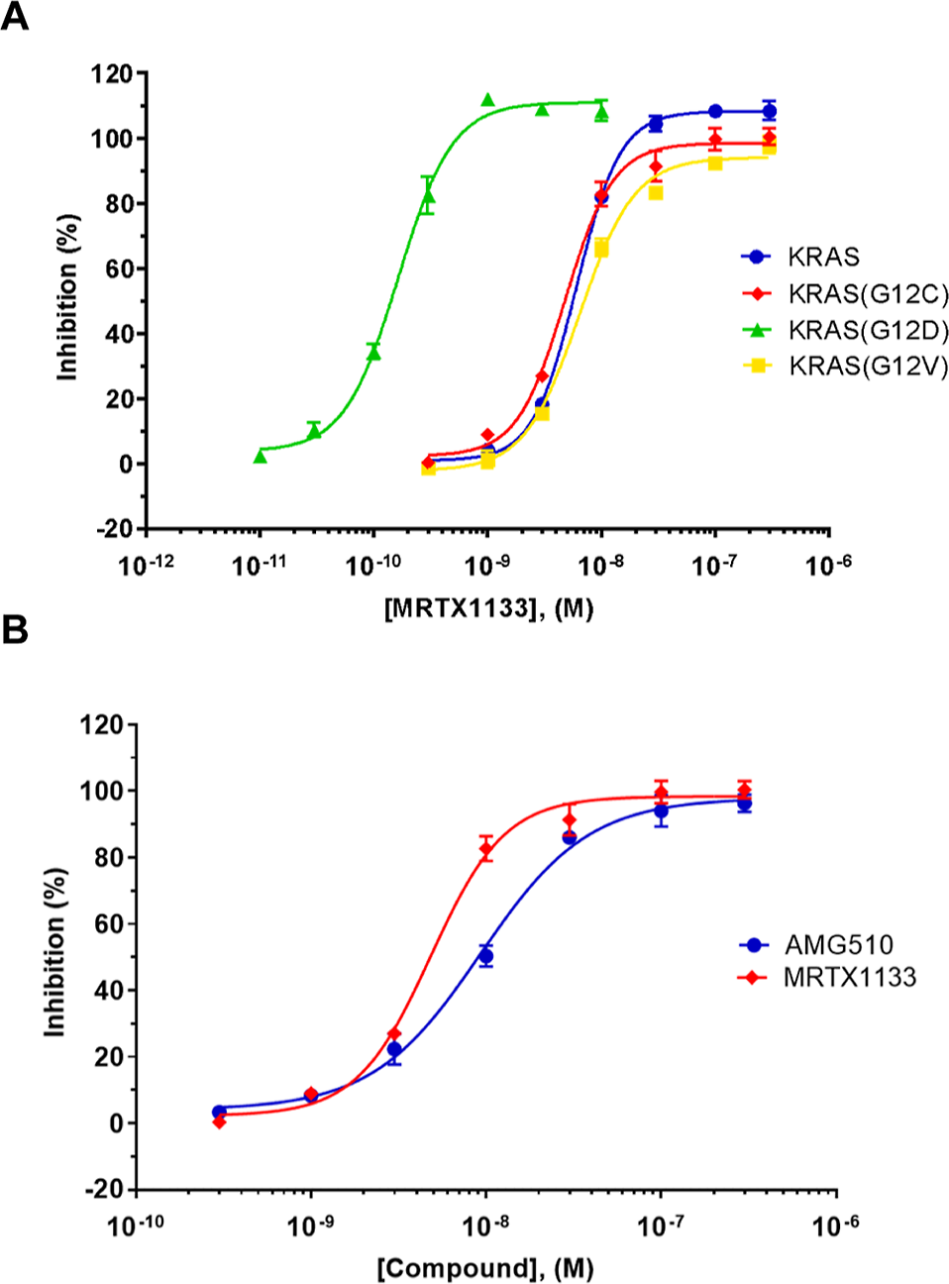
The inhibitory effects of MRTX1133 and AMG510 on nucleotide exchange against KRAS(WT) and mutants. (A) Relative potencies for MRTX1133 against KRAS(WT), KRAS(G12C), KRAS(G12D) and KRAS(G12V). (B) Inhibition potencies for MRTX1133 and AMG510 in the KRAS(G12C) assay. Error bars on data points represent the standard error of the mean for three independent experiments.

To confirm the affinity rank order profile for MRTX1133 and AMG510 that we observed in our developed biochemical binding assays, TR-FRET-based activity profiling of these compounds against KRAS(WT), KRAS(G12C), KRAS(G12D) and KRAS(G12V) was performed (Figure 4). The *Z*′ values were 0.71 (KRAS(WT)), 0.81 (KRAS(G12C)), 0.80 (KRAS(G12D)), and 0.85 (KRAS(G12V)), indicating the robustness of each biochemical activity assay. IC_50_ values for the inhibition of nucleotide exchange are shown in Table 2. Matching the compound affinity trend seen in the biochemical binding assays, MRTX1133 selectively inhibited KRAS(G12D) (IC_50_ = 0.14 nM) over KRAS(WT) (IC_50_ = 5.37 nM), KRAS(G12C) (IC_50_ = 4.91 nM) and KRAS(G12V) (IC_50_ = 7.64 nM). In addition, AMG510 inhibited KRAS(G12C) (IC_50_ = 8.88 nM), but no inhibition of KRAS(WT), KRAS(G12D) and KRAS(G12V) was observed up to 100 μM of compound; these results matched the relative affinity profile obtained for AMG510 in the biochemical binding assays.

**Table 2:** IC_5**0**_ Values of MRTX1133 and AMG510 against KRAS(WT) and Mutants^a^

protein	compound name	average IC_50_ (nM)
KRAS	MRTX1133	5.37 ± 0.22
	AMG510	ND
KRAS(G12C)	MRTX1133	4.91 ± 0.39
	AMG510	8.88 ± 1.17
KRAS(G12D)	MRTX1133	0.14 ± 0.01
	AMG510	ND
KRAS(G12V)	MRTX1133	7.64 ± 0.60
	AMG510	ND

a Average IC_50_ values are shown for replicates collected from three independent experiments ± standard error of the mean. ND: no inhibition detected at 100 μM compound.

### CRISPR-Mediated Knock-In

**Figure 5:**
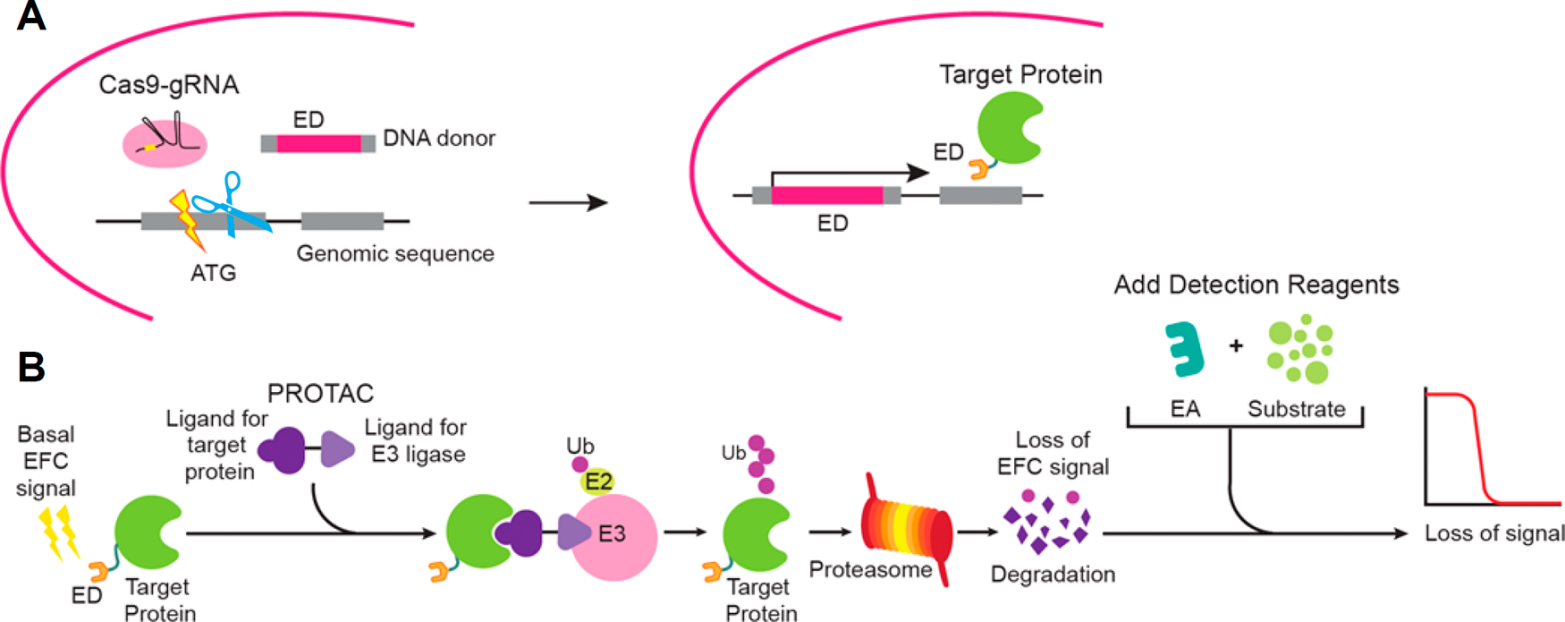
Schematic presentation of CRISPR-Cas9-mediated knock-in and the developed protein degradation assay using enzyme fragment complementation (EFC). (A) A KRAS cell line was generated by introducing a ribonucleoprotein complex containing gRNA and Cas9 protein along with a dsDNA donor comprised of the ED tag flanked by homologous recombination sequences into cells. Events leading to the in-frame knock-in needed to generate ED-target fusion proteins expressed at endogenous levels in a disease-relevant cell model are shown. (B) ED-target fusion proteins expressed at high or medium levels (basal EFC signal) are brought into proximity with an E3 ligase via a bifunctional molecule (such as a PROTAC). Subsequently, the target protein is ubiquitinated and degraded by the proteasome, resulting in a loss of EFC signal.

To capture the endogenous expression of KRAS in an A549 cell model, we introduced an ED tag (ePL or enhanced ProLink) into the *KRAS* locus to create an N-terminal tagged ED-KRAS fusion protein via the CRISPR-Cas9 system (Figure 5). The editing efficiencies of gRNAs that recognize sequences near the translation start site on *KRAS* exon 2 were first evaluated. gRNA (CR11.1) with an editing rate above 70% was used along with double-stranded DNA (dsDNA) donors that contained an ED tag flanked by homologous sequences with a G12C mutation or G12 to correct *KRAS*^*G12S*^ to *WT* in the A549 cells. The events of homologous recombination were detected by PCR-based molecular analysis and the occurrence of in-frame knock-in (KI) were confirmed by EFC activities. Cell pools underwent limiting dilution to isolate single-cell derived clones that harbored homozygous ED-*KRAS* alleles. We estimated that the homologous recombination frequencies were around 15–20%. Individual clones were further evaluated for their performance in functional assays, namely targeted protein degradation and target engagement. Best performing clones were tested for functional passage stability up to 15 passages and clones showing stable assay windows were used in the study. The KRAS(G12D) cell line was generated by introducing a G12D mutation into the established KRAS(G12C) line. Therefore, these two lines share virtually identical genetic backgrounds.

### Targeted Protein Degradation Assay

**Figure 6:**
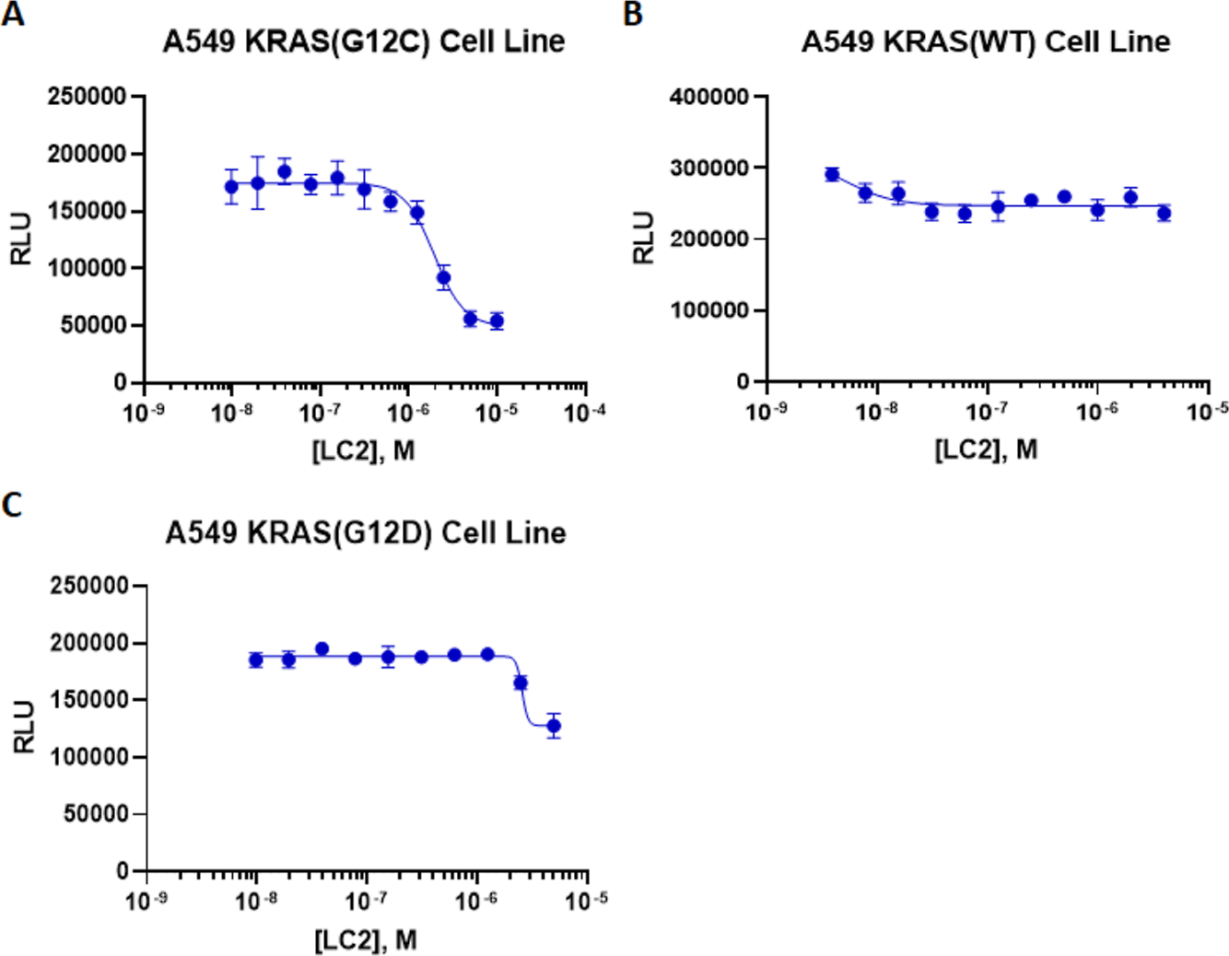
PROTAC LC2 selectively induces KRAS(G12C) protein degradation. Three KRAS cell lines (KRAS(G12C) (A), KRAS(WT) (B) and KRAS(G12D) (C)), were incubated with PROTAC LC2 for 18 h. LC2 selectively induces KRAS(G12C) protein degradation with a DC_50_ of 1.9 μM and *D*_max_ of 69% with an averaged *Z*′ of 0.72, yet spares the other KRAS proteins tested. Error bars on data points represent the standard deviation, *n* > 3 for KRAS(G12C), *n* = 3 for KRAS(WT) and KRAS(G12D). RLU = relative luminescence units.

We tested the KRAS(G12C)-specific protein degrader LC2 (Figure 1C) with our three KRAS cell lines in the developed protein degradation assay. The assay was set up in homogeneous 384-well format with an 11-point dose response curve, with a top dose of 10 μM and each dose tested in quadruplicate. Cells were incubated with LC2 for 18 h and at the end of incubation, detection reagents containing the complementing EA fragment were added to each well for chemiluminescence quantitation. Shown in Figure 6A, LC2 induced a loss of signal (an indication of protein degradation) in KRAS(G12C) cells with a DC_50_ of 1.9 μM and a *D*_max_ of 69%, close to the previously described value.^25^ The *Z*′ of 0.72 for the assay was generated from averaging three independent experiments and suggested that the assay was robust and could potentially be used for high throughput screening. In addition, protein degradation was only specifically induced in KRAS(G12C) but not in KRAS(WT). We observed some degradation in KRAS(G12D) cells at high doses of LC2(Figure 6A–C). This finding is consistent with reports that MRTX849 is specific to the KRAS(G12C) protein.^10^

### Pulse Target Engagement Assay

**Figure 7:**
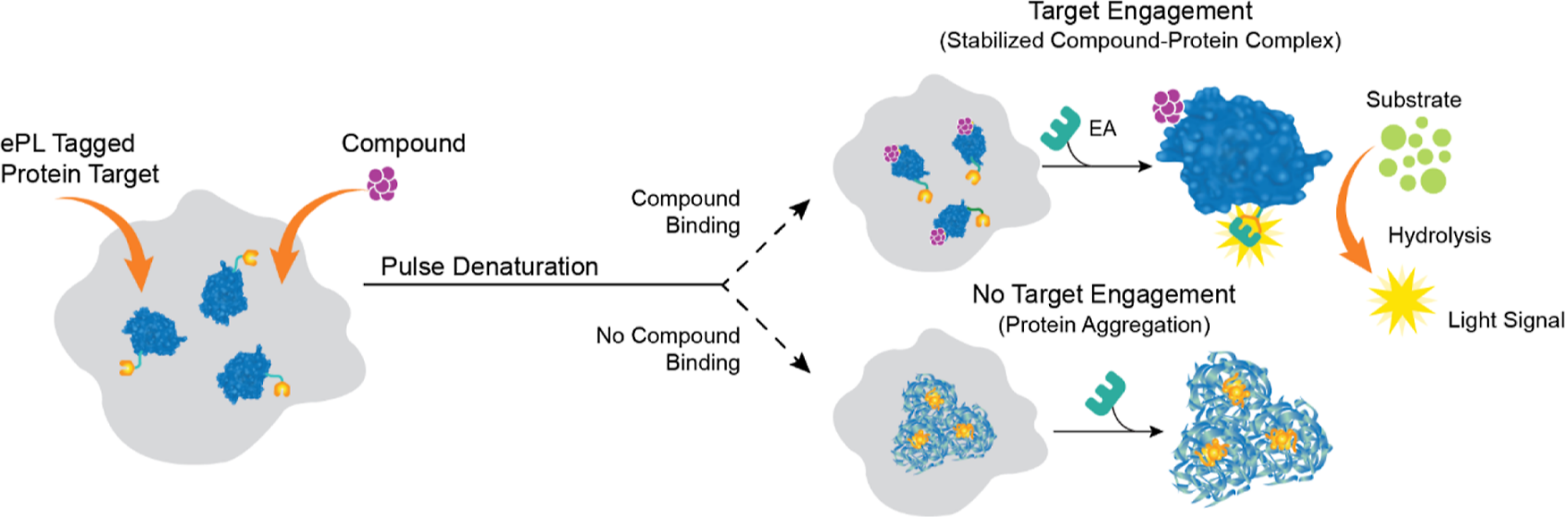
Graphical representation of the pulse target engagement assay. The assumption made for this target engagement assay is that a direct interaction between the target protein and a compound stabilizes the protein and reduces its turnover. Cells are incubated with a compound and then subjected to a pulse of an elevated temperature. Compound-bound target protein forms a heat-stable complex. Consequently, this preserves the EFC activity in the assay and increases the chemiluminescent signal. In the absence of compound binding, the protein is denatured, aggregates and loses its enzyme complementation ability, resulting in a low chemiluminescent signal.

Next, we explored the application of KRAS cell lines for a pulse target engagement (TE) assay to profile compounds or identify new ligands. The TE assay is based on the observation that the direct interaction between a compound and its cellular protein target increases the stability of the protein and reduces its turnover.^48,49,51^ Given that most endogenous proteins have a relatively long half-life, introduction of a thermal pulse has been demonstrated to induce protein turnover, increasing the success rate of TE assay development.^13,52^ Therefore, in our pulse TE assay, cells are incubated with a compound and then subjected to an elevated temperature to induce protein denaturation. Compound binding protects the target protein from denaturation, and consequently preserves enzyme complementation activities with the cellular ED-tagged fusion protein, producing a robust chemiluminescent signal. In the absence of compound binding, the target protein forms denatured aggregates that leads to a loss of complementation ability and decreased chemiluminescent signal (Figure 7).

**Figure 8:**
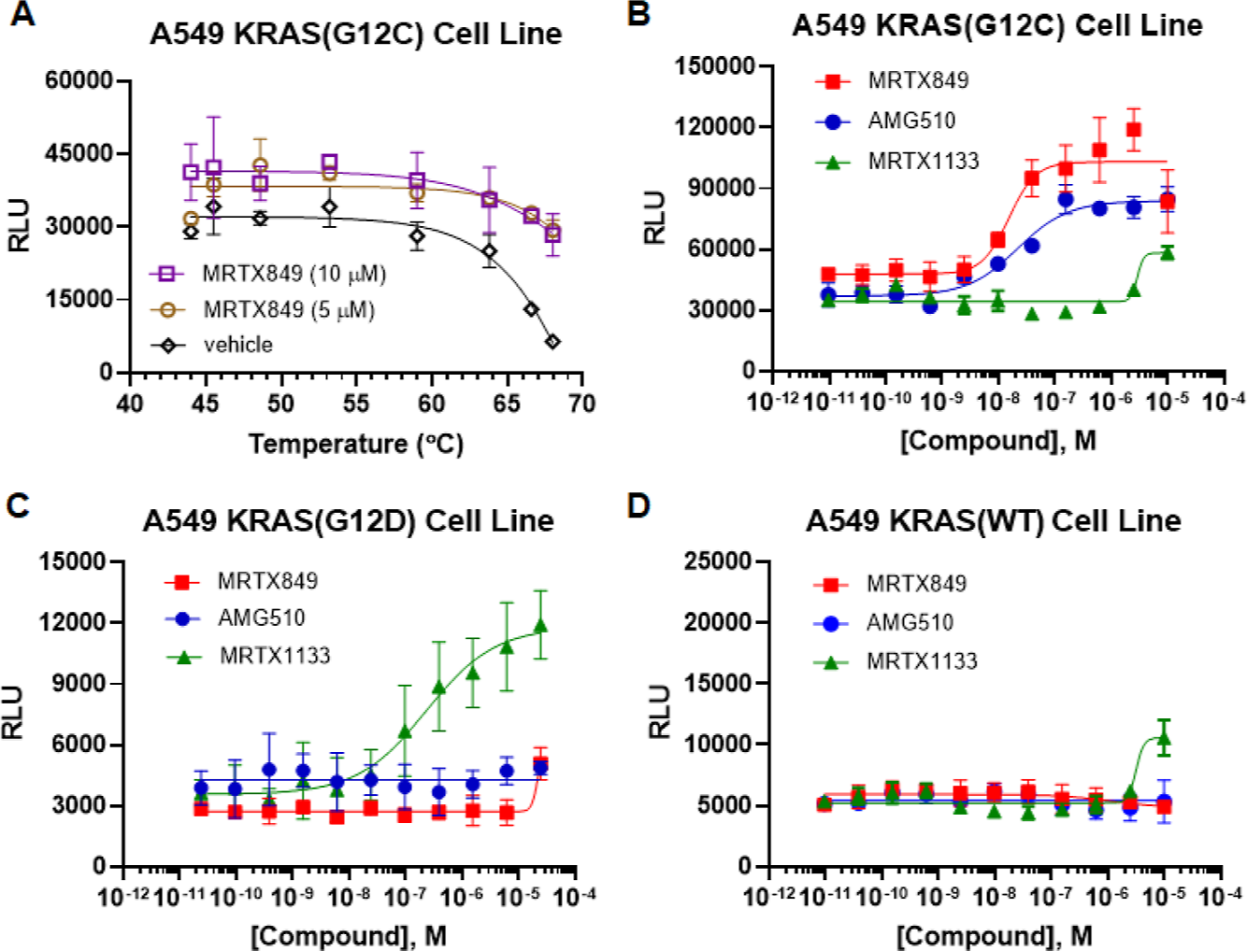
Developed target engagement assay reveals inhibitor selectivity to WT and mutant KRAS proteins. KRAS cell lines were used in target engagement assays to profile a panel of compounds including mutant specific inhibitors. (A) A thermal shift assay using the KRAS(G12C) cell line and its specific inhibitor MRTX849 was first performed to determine the pulse temperature for the target engagement assay. Cells were incubated at two different concentrations of MRTX849 or vehicle (1% DMSO) for 1 h, followed by a 3 min incubation at a gradient of elevated temperatures from 44 to 68 °C. A decrease in EFC activity appeared at temperatures above 60 °C and a substantial difference in EFC activity between MRTX849-treated and vehicle control-treated cells became most apparent around 66–68 °C. 67 °C was chosen as the pulse temperature for the target engagement assay. (B–D) For the target engagement assay, cells were incubated with compounds for 1 or 5 h followed by a 3 min incubation at 67 °C. MRTX849 and AMG510 showed high affinity to KRAS(G12C) protein (B). While MRTX1133 appears to have high selectivity for KRAS(G12D) protein (C), it also shows some interaction with KRAS(G12C) and KRAS(WT) protein (D). Error bars on data points represent the standard deviation, *n* = 5 for KRAS(G12C), *n* = 4 for KRAS(WT), *n* = 3 for KRAS(G12D). *Z*′ for KRAS(G12C) with MRTX849 is 0.36 and for KRAS(G12D) with MRTX1133 is 0.47.

First, a thermal shift assay was performed to determine the optimal pulse temperature for thermal insult in the pulse TE assay.^48^ KRAS(G12C) cells were treated with 10 and 5 μM of MRTX849 or vehicle control (1% DMSO) for 1 h, followed by a 3 min incubation at a gradient of elevated temperatures. Shown in Figure 8A, the KRAS protein appeared relatively heat-stable with a constant chemiluminescent signal detected up to a temperature of 60 °C. A thermal shift was observed at temperatures above 63 °C; in the presence of MRTX849, EFC activities remained steady while samples without the compound (vehicle control) had a significant drop in EFC activities. This finding indicates a direct interaction between KRAS(G12C) protein and MRTX849 as previously described, and this interaction protects the KRAS protein from thermal denaturation. We chose 67 °C as the pulse temperature since a roughly 50% difference in EFC activity was detected at this temperature between MRTX849-treated samples and the vehicle control, which would therefore provide a robust assay window.

We next performed pulse TE assays with a panel of KRAS inhibitors on three KRAS cell lines. MRTX849^10^ and AMG510^12^ are two inhibitors specific to the G12C mutant. MRTX1133 is a newly developed inhibitor that specifically targets the G12D mutant.^15^ Cells were incubated with the compounds for 1 or 5 h, followed by a 3 min incubation at 67 °C. Consistent with previous reports, we observed that MRTX849 and AMG510 appeared to have high affinity only for the KRAS(G12C) protein but not for KRAS(WT) or KRAS(G12D) proteins^10,12^ (*Z*′ = 0.36 with MRTX849) (Figure 8B–D). MRTX1133 showed high affinity for the KRAS(G12D) protein as previously described and some binding to KRAS(G12C) and KRAS(WT) proteins at high concentration (*Z*′ = 0.47 with MRTX1133).^15^ Despite that the robustness of these two assays is fair, these findings confirm that these KRAS cell lines are useful tools for compound profiling and could be used to determine the rank-order of new chemical matter when profiling compounds in dose response.

## Discussion

Major strides have been made in the development of KRAS mutant selective inhibitors in cancer, most notably for KRAS(G12C) and KRAS(G12D). Unfortunately, resistance to KRAS inhibitor monotherapy has been reported in the clinic;^53^ the basis for this resistance appears to be multifactorial: further mutation of KRAS which reduces inhibitor affinity for the target, changes in gene copy number, pathway rescue and alternative pathway utilization through mutation, transcriptional and epigenetic reprogramming and tumor microenvironment changes have all been proposed as mechanisms by which resistance is developed to this class of inhibitors.^54^ Combination therapy^55−58^ and next-generation inhibitors that evade resistance are being proposed as solutions to this issue. As such, development of next-generation KRAS inhibitors which evade resistance mechanisms or have a higher cellular fitness cost for the development of resistance are of importance to this field. Furthermore, the development of assay platforms that can identify selective inhibitors for mutants outside of G12C and G12D (for which multiple inhibitors have already been developed) will lead to a more tailored, personalized medicine approach for patients harboring other KRAS mutations prevalent in various cancers. Recent exciting advances in pan-KRAS mutant inhibitors, such as BI-2865,^22^ offer the potential for even broadly applicable treatment strategies for various mutant forms of KRAS. With the development of the new series of assays described in this paper, we have expanded the array of tools available for the development of next-generation, mutant-selective inhibitors of KRAS that circumvent existing resistance pathways. The present set of assays is amenable to expansion, including for additional KRAS mutants other than the G12C, G12D and G12V mutants described here.

We quantitatively determined the binding affinity constants for MRTX1133, AMG510 and MRTX849 across WT and G12C, G12D and G12V KRAS in biochemical assays in this work. The measured affinity constant for MRTX1133 was in the picomolar range for the G12D mutant protein we tested, consistent with the estimated high binding affinity reported previously.^15^ In addition, we demonstrated that MRTX1133 bound mutant KRAS with higher affinity than for WT protein, as reported for this compound.^15^ We also observed selective binding of AMG510 and MRTX849 to KRAS(G12C), as published for these compounds.^10,12^ The binding selectivity profiles for MRTX1133 and AMG510 were also consistent with the data we collected for these compounds in our nucleotide exchange assays. Since the biochemical assays have been validated with the aforementioned reference compounds, they can be used to interrogate novel chemical matter for binding to the switch II binding pocket of KRAS for the discovery of next-generation KRAS inhibitors.

Using our developed targeted protein degradation assay, we show that the PROTAC LC2 is capable of selectively degrading KRAS(G12C) in cells while sparing KRAS(WT) and showing low levels of KRAS(G12D) degradation only at higher concentrations of the degrader. The selectivity of this compound for KRAS(G12C) recapitulates published results.^25^ This assay can be used to profile novel PROTACs or molecular glues for mutant KRAS for the development of next-generation therapeutics for the treatment of mutant KRAS tumors.

In the cell-based target engagement assays, we were also able to reproduce published mutant-specific binding selectivity in our target engagement assays for three of the inhibitors tested in the biochemical system: MRTX1133 was selective for KRAS(G12D), and AMG510 and MRTX849 were selective for KRAS(G12C).^10,12,15^ These assays measure the effectiveness of the tested compounds in cells and provide important information for new drug testing that is complementary to the biochemical assays, such as evaluating compound diffusion through the cell membrane and engagement of compounds with the target expressed at physiological levels within cells.

In this work, we presented a cell-based platform for therapeutic discovery targeting KRAS mutants prevalent among many human cancers.^2−5^ We demonstrated that the KRAS(G12C) cell line is highly useful for the development of assays for other KRAS mutants: we were able to simply introduce the KRAS(G12D) mutation using CRISPR KI in the cell line described herein. This allows for direct comparison of profiling results for our different mutants using a virtually identical genetic background.

Each of the assays presented in this study fit into a comprehensive biochemical and cell-based characterization platform for the profiling of putative inhibitors and degraders against KRAS and its mutants. KRAS inhibitor or degrader pharmacological parameters can be established progressively in a screening cascade: compounds can be assessed for KRAS binding affinity and mutant selectivity using the biochemical competition assay in a high-throughput manner. Hits from screening in this assay can then be chemically optimized for improved binding affinity and/or mutant selectivity via a structure–activity-relationship (SAR) campaign. Either in parallel with or after binding profiling, hit molecules can be tested for their ability to inhibit nucleotide exchange in the functional biochemical assay. Compounds which show promise in both the biochemical binding and functional inhibition assays can then be profiled in the cell-based target engagement assay to assess cell permeability/stability of KRAS inhibitors in a cellular background. Lastly, heterobifunctional degrader molecules developed from KRAS-specific warheads (identified in the aforementioned biochemical binding and functional inhibition assays and the cellular target-engagement assays) chemically coupled to E3 ligase binders via variable linkers can be tested for their ability to degrade KRAS and its mutants via the ubiquitin-proteosome system in the developed protein turnover assays. Compounds can also be functionally screened for their ability to act as molecular glue degraders of KRAS using the protein turnover assay system.

In sum, we have presented a collection of biochemical and cell-based assays to study KRAS inhibitors and degraders. These assays can be used as part of a pipeline to identify and characterize the parameters for protein binding, inhibition of nucleotide exchange or targeted protein degradation across WT and mutant KRAS proteins of novel chemical entities.

## Methods

### Small Molecules and Nucleic Acids

MRTX1133, MRTX849 and AMG510 were purchased from MedChemExpress (Monmouth Junction, NJ) and Selleck Chemicals LLC (Houston, TX). The selective KRAS degrader LC2 was purchased from Bio-Techne Corporation (Minneapolis, MN). The biotinylated affinity probe (compound 1), which was used for the KRAS competition binding assays, was custom synthesized by SAI Life Sciences (Watertown, MA). The ^1^H NMR, LC–MS and HPLC data for compound 1 is provided in the Supporting Information section (Figures S1–S3). DMSO was purchased from Sigma-Aldrich (St. Louis, MO). The DNA probe containing the PCR amplicon used to tag the NFκB fusion domain was custom synthesized by Thermo Fisher Scientific (Waltham, MA).

### Protein Constructs and Protein Expression

Wild-type (WT) KRAS and G12C, G12D and G12V mutants (M1/R164, using NCBI entry NP_203524.1 as a reference sequence) were designed as N-terminal fusions with the DNA binding domain of NFκB (consisting of residues 35–36 fused to residues 41–359 (as described previously^50^), using UniProt entry P19838 as a reference). KRAS proteins were expressed via transient transfection in HEK293 cells. Protein extracts were harvested in M-PER extraction buffer (Pierce Biotechnology, Rockford, IL) supplemented with 150 mM NaCl, 10 mM DTT, Protease Inhibitor Cocktail Complete (Roche Diagnostics GmbH, Mannheim, Germany) and Phosphatase Inhibitor Cocktail Set II (Merck KGaA, Darmstadt, Germany) following the manufacturer’s guidelines.

### Cell Culture

Native A549 and SPRINTer A549 KRAS WT, G12C and G12D cell lines from Eurofins DiscoverX (Fremont, CA) were maintained in DMEM medium supplemented with 10% fetal bovine serum and 1× PSG from Thermo Fisher Scientific (Waltham, MA). Cultures were maintained with a routine of medium renewal 2 to 3 times per week. Freezing medium for cryopreservation is 95% complete growth medium with 5% DMSO.

### Competition Binding Assays

Competition binding assays were designed and developed as previously described for kinases.^30−33^

Preparation of liganded beads was performed as follows: the biotinylated affinity ligand (compound 1) was incubated with streptavidin-coated magnetic beads (Thermo Fisher Scientific, Waltham, MA) for 30 min at 25 °C. In order to remove the unbound affinity ligand and to reduce nonspecific binding of proteins in the cell lysate, the liganded beads were then blocked with excess biotin (125 nM) and washed with a blocking buffer containing SeaBlock (Pierce Biotechnology), 1% BSA and 0.05% Tween 20.

The binding reactions were prepared with the DNA tagged KRAS protein extract, beads loaded with the affinity ligand and the competitor test compounds in a binding buffer (1× PBS, 0.05% Tween 20, 10 mM DTT, 0.1% BSA, 2 mg/mL sheared salmon sperm DNA) in deep well, natural polypropylene 384-well plates, catalog number 784201 (Greiner Bio-One, Kremsmünster, Austria) in a final volume of 19.7 μL. No enzyme purification steps were performed on the protein extracts before adding them to the reaction mixture, and the protein extracts were diluted 10,000-fold in the final reaction mixture (the final DNA-tagged enzyme concentration was less than 0.1 nM). Binding assay mixtures were incubated at 25 °C with shaking for 24 h (WT KRAS assay) or 1 h (G12C, G12D and G12V KRAS assays). A 24 h incubation time for the WT assay was required to have appreciable competition of the test compound with the bait molecule so that a useable assay window could be obtained. After the incubation period, the affinity beads were extensively treated with a wash buffer (1× PBS, 0.05% Tween 20) to remove unbound protein from the protein lysate. Using an elution buffer (1× PBS, 0.05% Tween 20 and either 20 μM MRTX1133 (WT KRAS assay) or 1 μM MRTX1133 (G12C, G12D and G12V KRAS assays)), the beads were resuspended and incubated at 25 °C while shaking for a 30 min period. The concentration of WT or mutant KRAS in the eluates was then determined by quantitative PCR. *K*_D_ values for each competitor compound were determined using 11 serial 3-fold dilutions. For each assay, the affinity ligand concentration present on the magnetic beads was optimized to ensure that the true thermodynamic *K*_D_ values for competitor molecules were measured, as described in detail previously.^33^

### Data Analysis for Competitive Binding Assays

Binding constants (*K*_D_s) for each experiment were calculated using a standard dose–response curve fitting of the data using the Hill equation
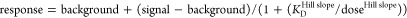


The Hill slope was set to −1. A nonlinear least-square fit using the Levenberg–Marquardt algorithm was employed for curve fitting.

### Nucleotide Exchange Assays

Biotinylated human recombinant KRAS WT, G12C, G12D, and G12V proteins (Amid Bioscience, Santa Clara, CA) were pretreated with terbium-labeled streptavidin (Tb-SA) (PerkinElmer, Waltham, MA) and BODIPY-FL-GDP (Thermo Fisher Scientific, Waltham, MA) overnight at room temperature in assay buffer containing 20 mM HEPES, pH 7.5, 50 mM NaCl, 10 mM MgCl_2_, and 0.01% (w/v) Tween 20. MRTX1133 and AMG510 were half-log diluted in duplicate and acoustically dispensed into 384-well plates (Corning, Corning, NY) by Echo 650 (Beckman Coulter, Brea, CA). Test articles were preincubated with pretreated KRAS/BODIPY-FL-GDP/Tb-SA for 1 h at 25 °C. To initiate the reaction, premixed SOS1 (Amid Bioscience, Santa Clara, CA) and unlabeled GTP (Sigma-Aldrich, St. Louis, MO) were added (total 20.2 μL per well) and incubated for 30 min at 25 °C. Final concentrations of Tb-SA, SOS1, and GTP were 0.17 nM, 50 nM, and 10 μM, respectively, for all assays. Final concentrations of KRAS proteins and BODIPY-FL-GDP were 10 and 100 nM, respectively, for the KRAS WT, G12C, and G12V assays, and were 1 and 40 nM, respectively, for the KRAS G12D assay. TR-FRET signals were obtained via excitation (337 nm) and emission (Tb: 486 nm and BODIPY: 515 nm) wavelengths using Infinite M1000 Pro spectrometer (TECAN, Männedorf, Switzerland). The inhibition percentages (%) for seven doses were analyzed and IC_50_ values were determined using GraphPad Prism (GraphPad Software, Boston, MA).

### CRISPR-Mediated Knock-In and KRAS Cell Line Generation

The gRNAs (crRNA), HiFi Cas9 Nuclease V3, and ATTO 550 tracrRNA used in this study to generate ED-tagged KRAS cell lines were purchased from Integrated DNA Technologies (Coralville, IA). Double-stranded DNAs containing ED or ePL (enhanced ProLink) were purchased from Eurofins Genomics Blue Heron (Bothell, WA). The gRNAs (crRNA) were designed using CRISPick, a design tool developed by the Broad Institute, with a CRISPRko mechanism by SpyoCas9 enzyme (*Streptococcus pyogenes*, NGG) on the Human Reference Genome GRCh38 (https://portals.broadinstitute.org/gppx/crispick/public). A gRNA (CR11.1, GAATATAAACTTGTGGTTGT) was used to generate KRAS(G12C) and KRAS(WT) cell lines. The gRNA was first annealed with fluorescence-labeled (ATTO-550) tracrRNA in a 1:1 ratio to form an RNA duplex, followed by incubation with Cas9 nuclease to generate Cas9-gRNA ribonucleoprotein (RNP). Cas9-gRNA RNP and double-stranded DNA (dsDNA) donors that contained ED fragments flanked by 120–180 bps of homologous sequences and the WT G12 sequence or G12C mutation were delivered into native A549 cells using the Neon Electroporation System from Thermo Fisher Scientific (Waltham, MA). At 16 h postelectroporation, cells with high fluorescence at 550 nm were sorted to enrich the genome-edited cell populations. PCR-based molecular analysis was performed to verify homologous recombination events. These cell pools underwent limiting dilution to generate single cell derived clones. Homozygous KI clones were identified by sequencing analysis and clones with the best assay windows for the protein degradation assay or the target engagement assay were chosen for this study. gRNA CR11.2 (GTAGTTGGAGCTGGTGGCGT) and a single-stranded DNA donor containing the G12D mutation were introduced into the KRAS(G12C) cell line to generate the KRAS(G12D) cell line following a similar protocol to the one described above.

### Targeted Protein Degradation Assay

Using the ED-tagged KRAS WT, G12C or G12D cell lines and the EFC detection kit (PathHunter ProLabel/ProLink Detection Kit, Eurofins DiscoverX, Fremont, CA), protein degradation assays were set up to test the potency of PROTAC LC2 in 384-well format in 11-point dose response (quadruplicate wells/dose), with the last well in each dose curve containing the vehicle control. Cells were detached from culture dishes with accutase (Innovative Cell Technologies, San Diego, CA) and collected in conical tubes. Spent medium and accutase were removed by centrifugation and the cells were resuspended in an appropriate amount of AssayComplete Cell Plating 0 Reagent (CP0, Eurofins DiscoverX, Fremont, CA) to allow a cell seeding of 5000 cells in 20 μL per well in a 384-well assay plate (white, clear flat bottom, Corning, Corning, NY). LC2 was diluted in CP0 to 50 μM (5× concentration for a top dose of 10 μM in the final assay) followed by 2-fold serial dilutions to generate the remainder of the 10 doses in the dose curve. Five μL of the 5× stock of LC2 was added to each well and 5 μL of CP0 was added to the vehicle control wells. The assay plates were placed in an incubator at 37 °C with 5% CO_2_ for 18 h. Working detection reagent solution was prepared by mixing a 4:1:1 ratio of the ED (PL/PK) detection reagent as described in the manufacturer’s user manual. Thirty μL of the working detection reagent solution was added to each well at the end of compound incubation and the plates were left in the dark at room temperature for 1 h. Plates were read using an Envision plate reader (PerkinElmer, Shelton, CT) using a 0.1 s/well integration time. The dose–response curves were generated using GraphPad Prism with [inhibitors] vs response—variable slope. DC_50_, drawn from best–fit curve, is the concentration of degrader that gives a half-maximal degradation response. *D*_max_ is determined as previously described.^59^*Z*′ is calculated by using the readings of the top dose as the positive control and the readings of vehicle as the negative control.

### Pulse Target Engagement Assay

This workflow was adapted from the InCELL Pulse Target Engagement Assay (Eurofins DiscoverX, Fremont, CA). A thermal shift assay was first performed to determine the optimal temperature for thermal insult in the pulse target engagement assay. KRAS(G12C) cells were collected and diluted in complete medium that allowed for a cell seeding of 5000 cells in 40 μL per well in an 8-point dose curve (triplicate wells per dose) in 96-well PCR plates (black, Thermo Fisher Scientific, Waltham, MA). Working stocks (5×) of MRTX849 (50 and 25 μM) were made in complete medium with 5% DMSO. Ten μL of MRTX849 working stock (5×) was added to each well to generate final concentrations of 10 and 5 μM with 1% DMSO. For vehicle control samples, 10 μL of 5% DMSO in complete medium was added to each well. The plates were placed in an incubator at 37 °C, 5% CO_2_ for 1 h, followed by a 3 min incubation at a gradient of elevated temperatures between 44 and 68 °C in a C1000 Touch Thermal Cycler (BioRad Life Science, Hercules, CA). The plates were then cooled at 25 °C for 3 min and 60 μL of the working detection reagent solution (ProLabel/ProLinK Detection Kit, Eurofins DiscoverX, Fremont, CA) described above was added to each well at the end of the cool down. The plates were left in the dark at room temperature for 1 h before reading chemiluminescence signal on an EnVision plate reader.

The pulse target engagement assay was set up in a 96-well format with an 11-point dose response curve (triplicates/dose) and vehicle control. Cells were collected and diluted in complete medium that allowed for a cell seeding of 5000 cells in 40 μL per well in 96-well PCR plates (black, Thermo Fisher Scientific, Waltham, MA). Compounds were diluted in complete medium to 125 μM (5× concentration for a top dose of 25 μM in 5% DMSO) followed by a 4-fold serial dilution to make the rest of the 10 doses. Ten μL of 5× stocks of serially diluted compounds were added to each well in the assay plate and 10 μL of complete medium was added to the vehicle control well. The assay plates were placed in an incubator at 37 °C, 5% CO_2_ for 1 or 5 h. The plates were placed in a thermal cycler for a 3 min incubation at 67 °C, followed by another 3 min incubation at 25 °C to cool down. Sixty μL of the working detection reagent solution (same as above) was added to each well and the plates were left in the dark at room temperature for 1 h before reading chemiluminescence signal using an EnVision plate reader.

## Supporting Information Available

^1^H NMR, LC–MS and HPLC data for compound 1, Figures S1–S3 (PDF)
