# Supplementary material for: Suite of Biochemical and Cell-Based Assays for the Characterization of Kirsten Rat Sarcoma (KRAS) Inhibitors and Degraders
Source: ACS Pharmacol Transl Sci. 2024 Dec 2;7(12):3921–34. doi: 10.1021/acsptsci.4c00450 (PMC11651172; doi:10.1021/acsptsci.4c00450)
Supplement: Supplementary file 1 — pt4c00450_si_001.pdf [file pt4c00450_si_001.pdf]

SUPPORTING INFORMATION

Suite of Biochemical and Cell-Based Assays for the Characterization of Kirsten Rat Sarcoma (KRAS) Inhibitors and Degraders

Medhanie Kidane[1#], Rene M. Hoffman[2#], Jennifer K. Wolfe-Demarco[1#], Ting-Yu Huang[3], Chi-Ling Teng[3], Saheli Samanta[2], Luis M. Gonzalez Lira[1], Jennifer Lin-Jones[2], Gabriel Pallares[1], Jane E. Lamerdin[2], Nicole B. Servant[1], Chun-Yao Lee[3], Chao-Tsung Yang[2*], Jean A. Bernatchez[1*]

[1]Research and Development and Technology Transfer, Eurofins DiscoverX, LLC, 11180 Roselle Street, Suite D, San Diego, CA 92121, United States of America

[2]Research and Development, Eurofins DiscoverX Products, LLC, 42501 Albrae Street, Fremont, CA 94538, United States of America

[3]Eurofins Panlabs Discovery Services Taiwan, Ltd., 25 Wugong 6th Road, Wugu District, New Taipei City 24891, Taiwan

[#]These authors contributed equally.

[*]To whom correspondence should be addressed: Chao-Tsung Yang, e-mail address: Chao-Tsung.Yang@discovery.eurofinsus.com, Jean A. Bernatchez, e-mail address: Jean.Bernatchez@discovery.eurofinsus.com

DIS-379-106731-001-102-2{ATMEDU22023-218043} in MeOD
FILE NO:BNM02110723BM054
BRUKER 400MHz

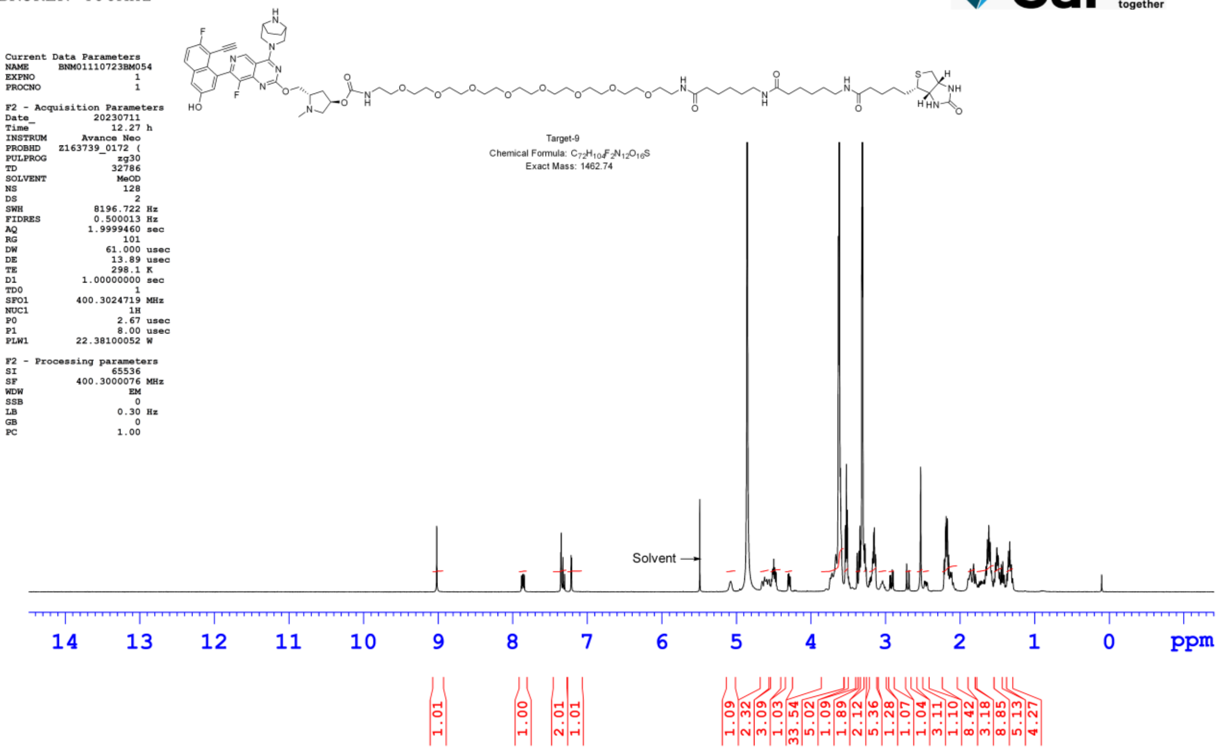

DIS-379-106731-001-102-2{ATMEDU22023-218043} in MeOD
FILE NO:BNM02110723BM054
BRUKER 400MHz

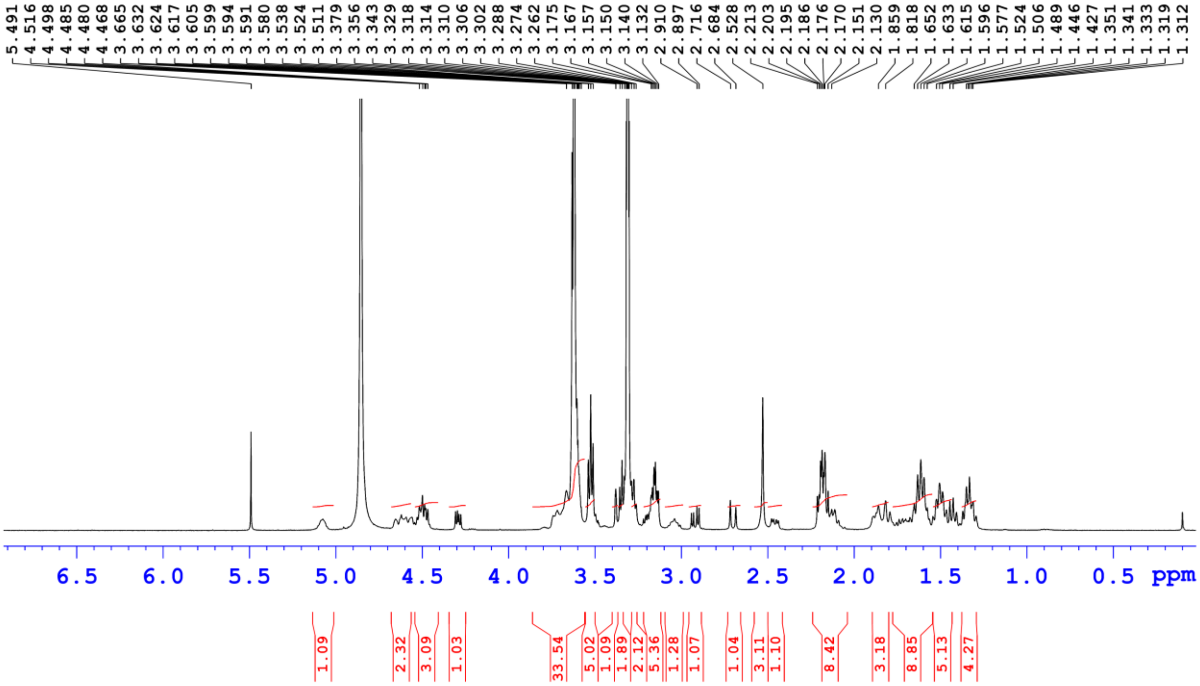

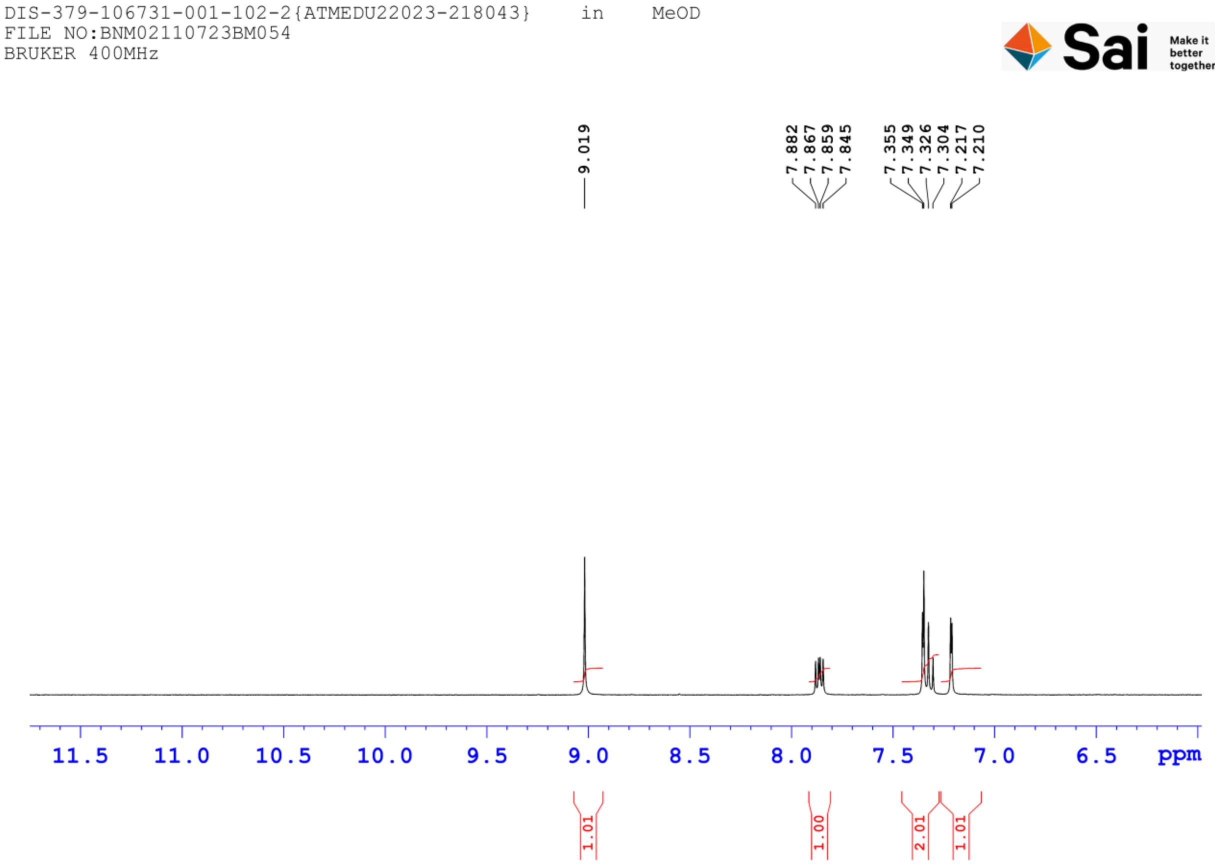

Supplemental Figure S1. ^{1}NMR data for compound 1, the capture ligand for the biochemical competition binding assay for WT and mutant KRAS.

SAMPLE ID:DIS-379-106731-001-102-2
TRF:ATMEDU22023-218042
Vial NO:1:C,3

Sai life Sciences Private limited
Analytical Medchem Division

Date of Analysis:11-Jul-2023;08:51:35
Instrument ID:AMC-LCMS-16

11072023_021A

3: Diode Array
Range: 4.61e+1

Time	Height	Area	Area%
1.59	45905672	1450141.13	97.84
1.69	375511	12225.38	0.82
1.89	110275	1660.37	0.11
2.52	541517	9023.07	0.61
2.83	531575	9107.67	0.61

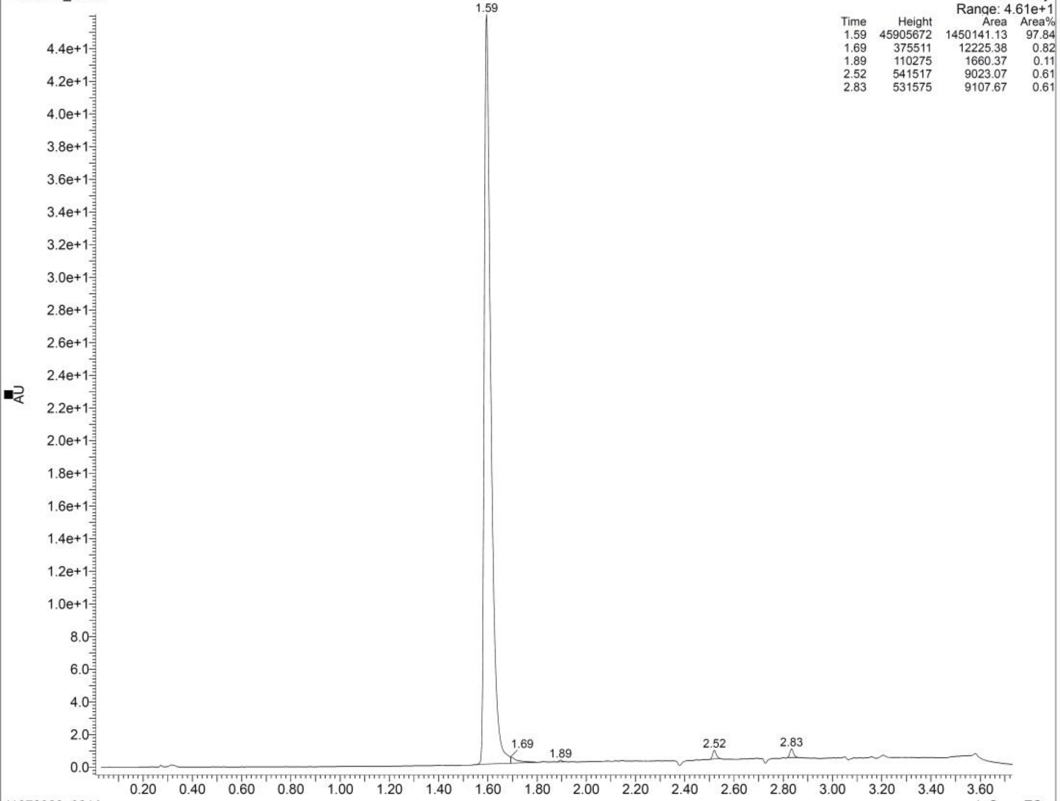

11072023_021A

1: Scan ES+
TIC
8.11e8

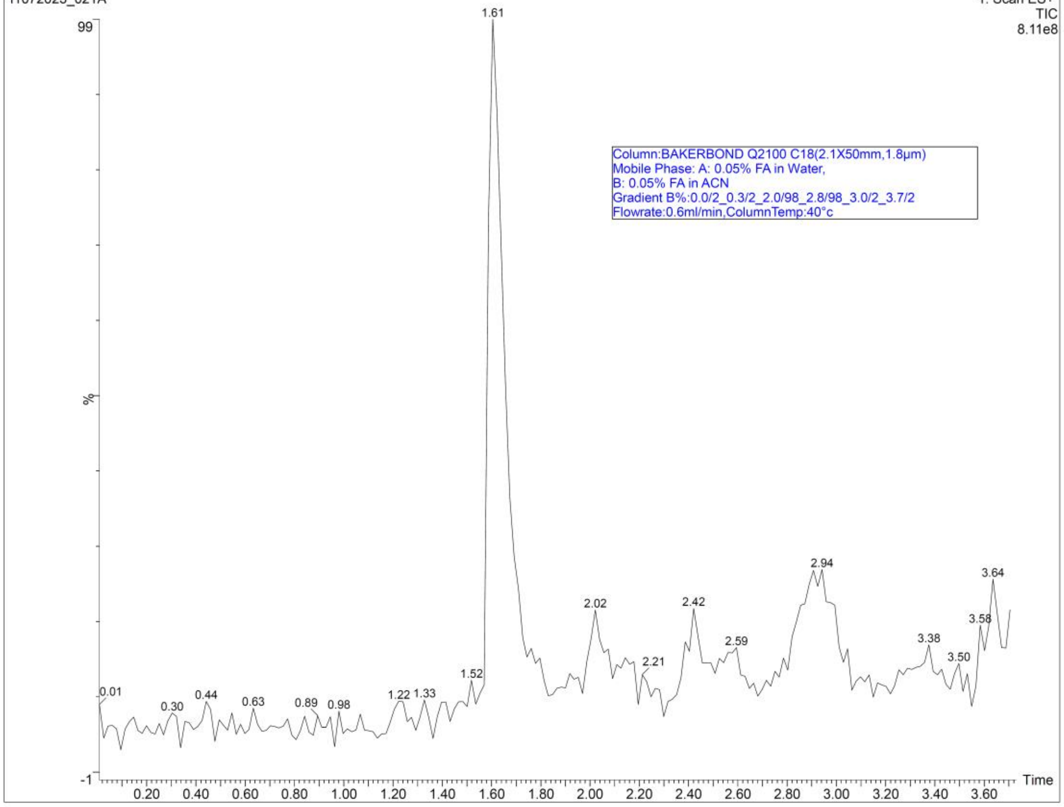

Column:BAKERBOND Q2100 C18(2.1X50mm,1.8µm)
Mobile Phase: A: 0.05% FA in Water,
B: 0.05% FA in ACN
Gradient B%:0.0/2_0.3/2_2.0/98_2.8/98_3.0/2_3.7/2
Flowrate:0.6ml/min,ColumnTemp:40°c

SAMPLE ID:DIS-379-106731-001-102-2
TRF NO:ATMEDU22023-218042
Vial NO:1:C,3

Date of Analysis:11-Jul-2023;08:51:35
Instrument ID:AMC-LCMS-16

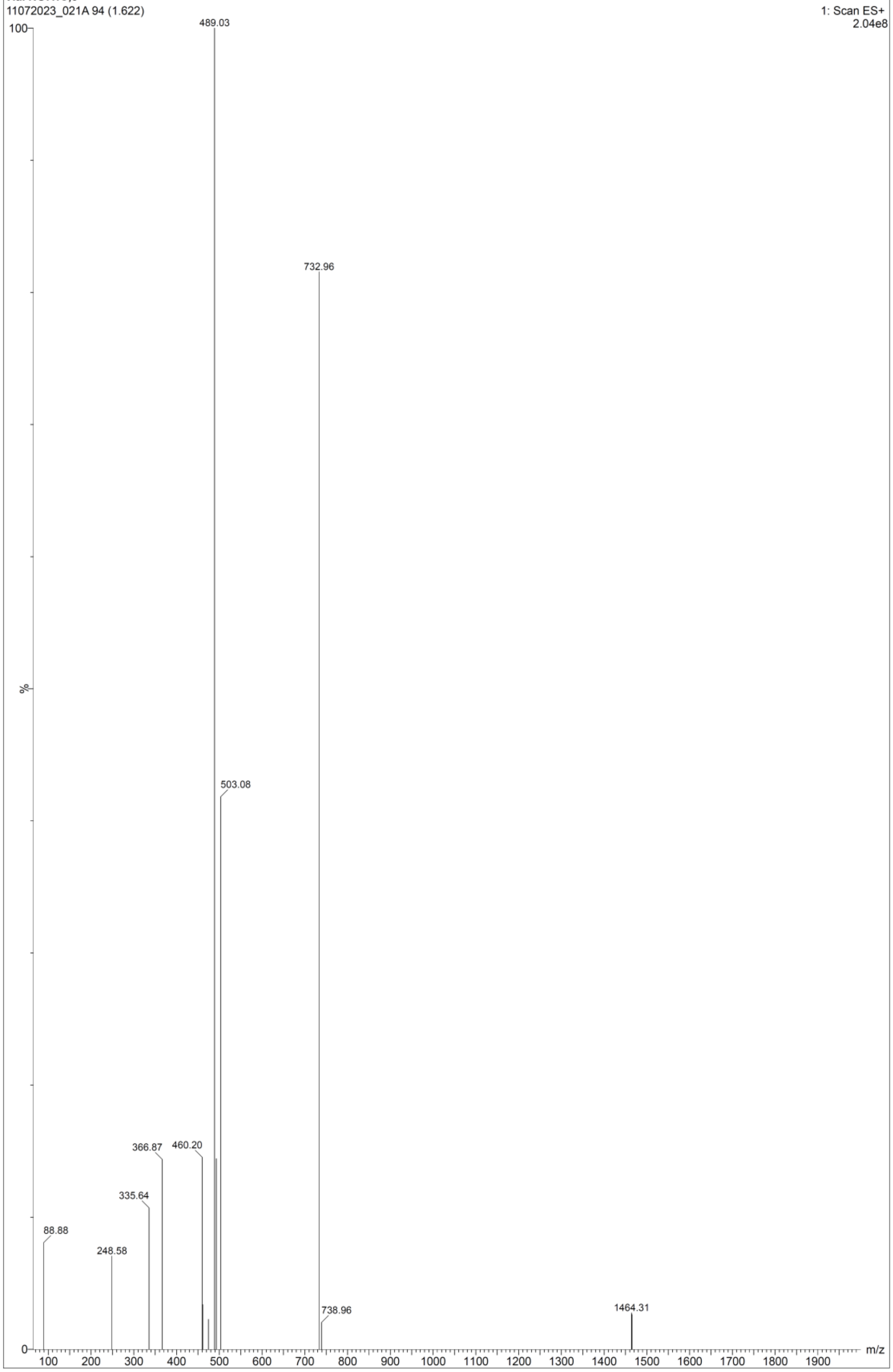

SAMPLE ID:DIS-379-106731-001-102-2
TRF NO:ATMEDU22023-218042
Vial NO:1:C,3

Date of Analysis:11-Jul-2023;08:51:35
Instrument ID:AMC-LCMS-16

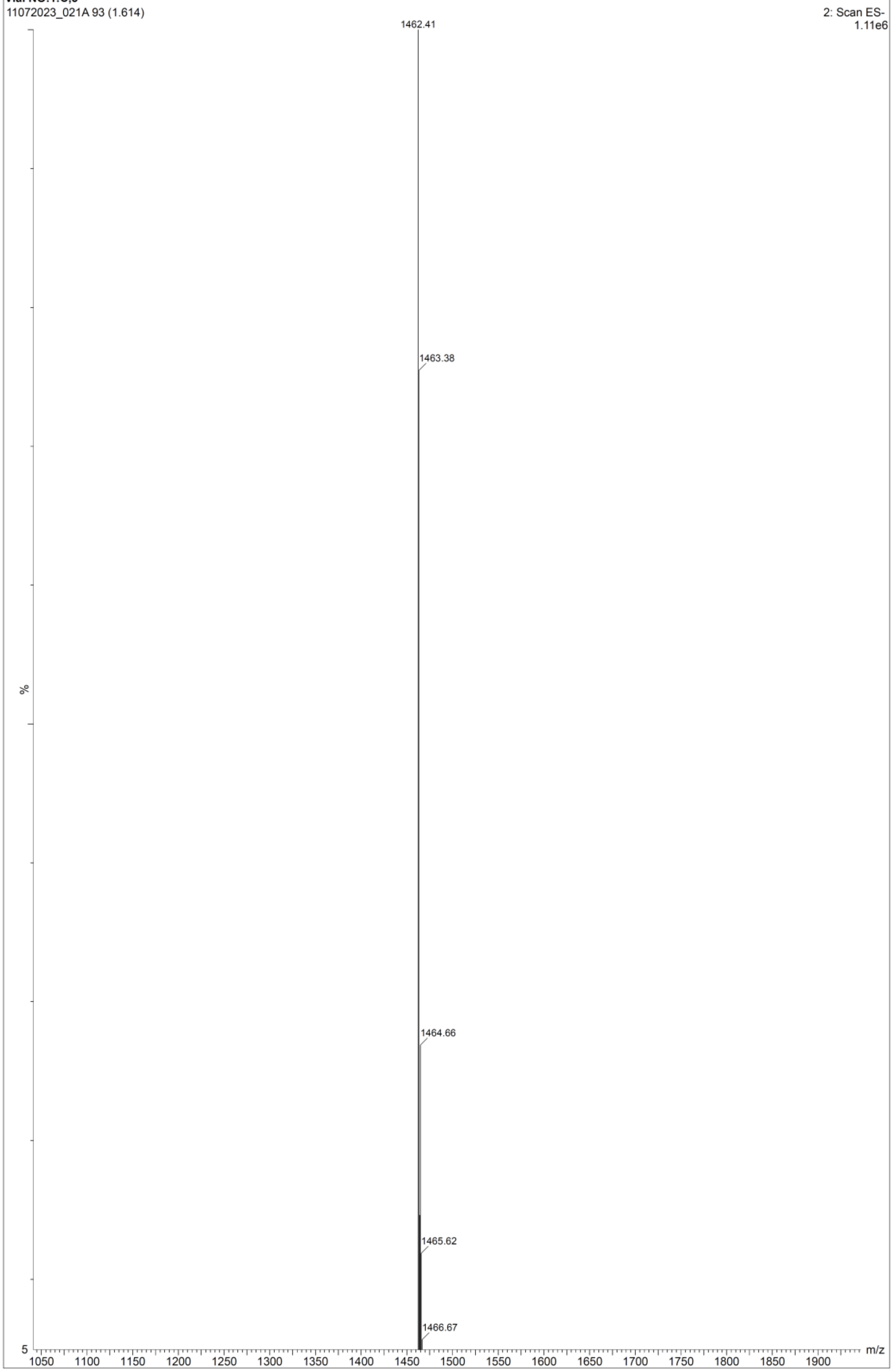

Supplemental Figure S2. LC-MS data for compound 1, the capture ligand for the biochemical competition binding assay for WT and mutant KRAS.

HPLC REPORT

Sample Information

Acquired by : Admin
HPLC ID : AMC-HPLC-12
Date Acquired : 7/11/2023 11:10:44 AM
Inj Volume : 6 µL
Sample Name : LCMS
Sample ID : DIS-379-106731-001-102-2
Data File : 015.lcd
Method File : ABC_REG_18min_2023.lcm
AR number : ATMEDU22023-218041

Method:- HPLC_X-Bridge
Column : X-Bridge C18 (4.6*150) mm 5.0 µm
Mobile Phase: A - 5 mM ABC in Water
B - Acetonitrile
Flow Rate: 1.0 mL/minute
Gradient program: Time(min)/ B Conc. :

0.01	Pumps	Pump B Conc.	5
1.00	Pumps	Pump B Conc.	5
8.00	Pumps	Pump B Conc.	100
12.00	Pumps	Pump B Conc.	100
14.00	Pumps	Pump B Conc.	5
18.00	Pumps	Pump B Conc.	5
18.01	Controller	Stop	

Chromatogram

LCMS F:\new instrument\AMC-HPLC-12\2023\DATA\JULY-2023\11\samples\015.lcd

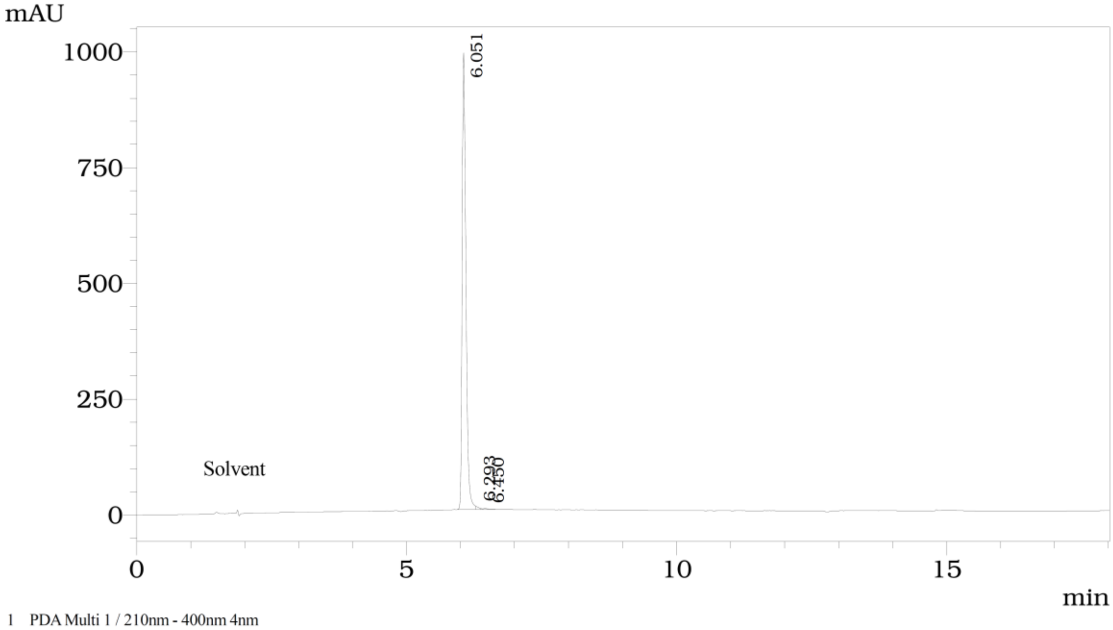

1 PDA Multi 1 / 210nm - 400nm 4nm

PeakTable
PDA Ch1 210nm - 400nm 4nm

Peak#	Ret. Time	Area	Area %	Peak Purity Index
1	6.051	4636624	99.273	1.00000
2	6.293	19872	0.425	0.99987
3	6.450	14060	0.301	0.99268

Peak#	Ret. Time	Area	Area %	Peak Purity Index
Total		4670556	100.000	

Supplemental Figure S3. HPLC data for compound 1, the capture ligand for the biochemical competition binding assay for WT and mutant KRAS.
